# Tuberculosis contact investigations associated with air travel in Ireland, September 2011 to November 2014

**DOI:** 10.2807/1560-7917.ES.2016.21.40.30358

**Published:** 2016-10-06

**Authors:** Paula Flanagan, Joan O'Donnell, Jolita Mereckiene, Darina O'Flanagan

**Affiliations:** 1 Health Protection Surveillance Centre, Dublin, Ireland

**Keywords:** Tuberculosis, *M. tuberculosis*, contact tracing, air travel

## Abstract

The risk of communicable disease transmission during air travel is of public health concern and has received much attention over the years. We retrospectively reviewed information from nine flights (≥ 8 hours) associated with infectious tuberculosis (TB) cases in Ireland between September 2011 and November 2014 to investigate whether possible transmission had occurred. Twenty-four flights notified in Ireland associated with sputum smear-positive pulmonary TB cases with a history of air travel were reviewed. Nine were suitable for inclusion and analysed. Six cases of infectious TB travelled on nine flights. A total of 232 passengers were identified for contact tracing; 85.3% (n = 198) had sufficient information available for follow-up. In total, 12.1% (n = 24) were reported as screened for TB. The results revealed no active TB cases among passengers and 16.7% (n = 4) were diagnosed with latent TB infection (LTBI) all of whom had other risk factors. Despite the limited sample size, we found no evidence of *M. tuberculosis* transmission from infectious passengers. This study identified challenges in obtaining complete timely airline manifests, leading to inadequate passenger information for follow-up. Receipt of TB screening results from international colleagues was also problematic. The challenge of interpreting the tuberculin skin test results in determining recent vs earlier infection was encountered.

## Introduction

Tuberculosis (TB) remains a significant cause of mortality and morbidity with an estimated 9.6 million new TB cases reported worldwide each year [1]. Drug resistance is also a major challenge with 3.3% of new TB cases and 20% of previously treated cases having multidrug-resistant TB (MDR-TB) globally [1]. Although great progress has been made in the control of TB in recent years, it remains a public health concern in most countries in the World Health Organization (WHO) European Region with an estimated 360,000 incident TB cases occurring during 2013 [2]. The absolute number of incident TB cases fell by 20,000 in 2013, corresponding to a 5.6% decline compared with the previous year in Europe [2].

In Ireland the incidence of TB has been declining. Over the past 10 years, the number of TB cases notified decreased from 450 in 2005 to 318 cases in 2014 [3].

As TB remains a serious global public health issue, many interventions are aimed at preventing and controlling disease transmission nationally and internationally. Contact tracing is one of the key measures in the management and control of TB as early detection of new cases reduces the timeframe during which a person is infectious.

Many studies have been conducted to investigate the possibility of TB transmission during air travel. The Centers for Disease Control and Prevention (CDC) in the United States (US) conducted six investigations between 1992 and 1995 examining the possible transmission of TB during air travel [4]. Only two of these investigations reported evidence of possible TB transmission [5,6]. Other studies, including an extensive systematic review conducted in the United Kingdom (UK), found the risk of transmission to be low or inconclusive [7-13].

The length of contact necessary for TB infection to be transmitted is variable and depends on a number of factors including the infectiousness of the index case, the susceptibility of the individual exposed and the environment where the exposure occurred [14]. Guidelines published by WHO on TB and air travel [15] state that the risk of possible TB disease transmission during air travel is associated with sitting within two rows of an infectious passenger on flights lasting 8 hours or longer. The guidelines also recommend contact tracing be conducted within the 3-month period between date of travel and date of notification. Given the difficulties in assessing infectiousness at the time of the flight, interpreting tuberculin skin test (TST) results to determine recent vs earlier infection and obtaining sufficient accurate passenger travel and seating details, 3 months is considered the maximum time after travel that warrants public health intervention [15].

In Ireland, the 2010 guidelines on the prevention and control of tuberculosis [16] recommend contact tracing for passengers on board an aircraft who were exposed to a confirmed case of infectious TB as per the WHO guidelines. In this context, we decided to review information on all cases of infectious TB associated with air travel reported in Ireland between September 2011 and November 2014 to investigate the possibility of TB transmission.

## Methods

All TB notification records were reviewed to identify cases with a history of air travel. Flights lasting less than 8 hours as well as flights where the 3-month period had elapsed between the date of the flight and the date of notification to public health authorities were excluded from the analysis. All cases of sputum smear-positive pulmonary TB with a history of air travel on flights of 8 hours or more duration in the 3 months before notification to the Health Protection Surveillance Centre (HPSC) in Ireland between September 2011 and November 2014 were retrospectively reviewed. The following outlines the steps taken during the investigation of flight contacts.

For each case notified to HPSC, data were collected on the index case from the notifying clinician on the site of disease, symptoms including onset date, treatment and microbiology results including drug sensitivities where available. Details of the relevant flights were obtained from regional public health departments. Following this, the relevant airline was contacted using a standardised letter and the passenger manifest requested as per national and WHO guidance on passenger contacts seated in the same row and two rows in front of and behind the index case in order to identify passengers requiring TB screening. Specific ethical approval was not required to undertake this study, as under the Irish Infectious Disease Regulations (1981) [17] follow-up of contacts of infectious cases of TB is required as part of routine work to prevent further spread of disease.

Where sufficient passenger contact information was available, this information was then sent to the relevant regional departments of public health in Ireland and internationally to the relevant national TB surveillance and control focal points and TB screening including results was requested on the contacts.

Data were analysed using case counts and frequencies.

## Results

Between September 2011 and November 2014, a total of 24 commercial flights associated with infectious cases of TB were reported in Ireland. Contact investigation was carried out on nine of these flights. Fifteen flights were not followed up: for five of these the 3-month period had elapsed between the date of the flight and the date of notification; and the airline manifest was not provided by the airline for seven flights, despite frequent requests. The remaining three flights were less than 8 hours duration and therefore no further follow-up was required.

For the nine flights investigated, the median estimated duration of flights was 8 h 40 min (range: 8 h to 11 h 40 min; IQR: 8 h 20 min to 8 h 40 min). A total of six index cases (four male, two female; age range: 33–81 years) travelled on the nine flights. All cases were diagnosed as sputum smear-positive pulmonary TB and were deemed to be infectious at the time of travel. The quality of the data received from the airline manifest varied between flights and airlines. Four of the index cases were diagnosed with pan-sensitive strains of *Mycobacteriumtuberculosis* and two index cases were diagnosed with *M*. *tuberculosis* resistant to isoniazid (Table).

**Table:** Tuberculosis contact investigations associated with air travel in Ireland, profile of index case, flight details, contact details and results, September 2011 to November 2014

Flight (n = 9)	Date of flight	Infectivity and diagnosis of index case (n = 6)	Age group (years) of index case at time of travel	Drug resistance	Number of potentially exposed passengers as per airline manifest^a^	Number of passengers with information available from airline manifest	Number of passengers with screening results available	Number of LTBI positives	Estimated duration of flight (h:min)	Interval (days) between date of flight and date of notification	Interval (days) between date of information request from airline and receipt of airline manifest
Dubai–Dublin	Nov 2014	Sputum smear-positive for *M. Tuberculosis*	50–59	Isoniazid	20	20	3^b^	0	8:20	41	3
Johannesburg–Dubai	Nov 2014				24	22	1^c^	0	8:00	42	3
Abu Dhabi–Dublin	Sep 2014	Sputum-smear positive for *M. Tuberculosis*	Unknown	No	15	6	2^d^	1	8:40	32	26
Rio de Janeiro–London	Sep 2013	Sputum smear-positive for *M. Tuberculosis*	30–39	Isoniazid	39	39	0	0	11:40	17	3
Abu Dhabi–Dublin	Jul 2013	Sputum smear-positive for *M. Tuberculosis*	40–45	No	27	18	6^e^	0	8:40	56	36
Johannesburg–Abu Dhabi	Jul 2013				10	7	0	0	8:20	23	36
Abu Dhabi–Johannesburg	Jun 2013				27	16	3^f^	0	8:40	55	36
Dubai–Dublin	Jul 2012	Sputum smear-positive for *M. Tuberculosis*	80–89	No	26	26	9^g^	3	8:15	9	2
Paris–Seoul.	Sep 2011	Sputum smear-positive for *M. Tuberculosis*	30–39	No	44	44	0	0	10:50	95	6
**Total**	NA		NA	NA	**232**	**198 (85.3%)**	**24 (12.2%)**	**4 (16.6%)**	NA	NA	NA
**Median (interquartile range)**	NA		NA	NA	NA	NA	NA	NA	**8:40 (8:20–8:40)**	**41 (23–45)**	**6 (3–36)**

CXR: chest X-ray; IGRA: interferon-gamma release assay; LTBI: latent tuberculosis infection; *M. tuberculosis: Mycobacterium tuberculosis*; NA: not applicable; TST: tuberculin skin test.

^a^Passengers seated in the same row and two rows in front of and behind the index case.

^b^One contact had TST and two contacts had IGRA only performed.

^c^One contact had TST only performed.

^d^IGRA performed on both contacts.

^e^Five contacts had TST only performed; one contact had TST and IGRA.

^f^Two contacts had TST only performed and one contact had was clinically assessed for active tuberculosis.

^g^Four contacts had TST only performed; one contact had a CXR only; two contacts had both a TST and CXR; one contact had an IGRA; one contact had an IGRA and CXR.

Of the nine flights investigated a total of 232 passengers were identified for TB contact tracing. Of these identified passengers, 85.3% (n = 198) had sufficient personally identifiable information available from the airline manifest. No airline crew were included for contact tracing. The number of passengers requiring TB screening on each flight varied due to the type of aircraft and whether a bulkhead wall was situated within the five rows that were relevant for contact tracing.

Follow-up was made with local and international colleagues for TB screening results. Screening results were reported for six of the nine flights.

Where information requesting TB screening on passenger contacts was available, 10.6% (n = 21) were Irish citizens, and 89.4% (n = 177) were international contacts. Screening results were obtained on a total of 24 passenger contacts. Of these 24 passenger contacts with screening results obtained, 16 were screened in Ireland and eight were screened abroad. Two passenger contacts were identified as family members and deemed close contacts to one of the index cases; no other relationships were identified between the index cases and other passenger contacts.

Where TB screening results were available (n = 24) the type of test used for screening was available for 23 passengers. One passenger was clinically assessed for active TB and was from a country of high endemicity (≥ 40 cases of TB per 100,000 population per year).

**Figure 1:**
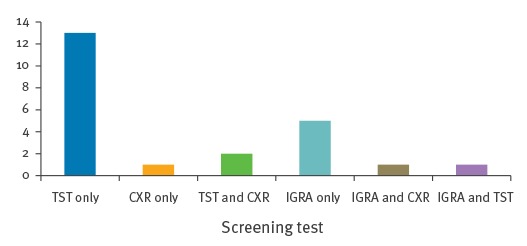
Tuberculosis screening test type used in contact investigations associated with air travel in Ireland, September 2011 to November 2014 (n = 23)

CXR: chest X-ray; IGRA: interferon-gamma release assay; TST: tuberculin skin test.

Figure 1 below presents details of the type of test used for TB screening in the 23 passengers where information on type of screening test used was available. The majority of passenger contacts were screened using the tuberculin skin test (TST). Information on the size of the TST induration was not available for most of the passengers and results were reported as being positive or negative.

One passenger was clinically assessed for active TB only, was resident in a high-endemic country and is not presented in this figure.

**Figure 2:**
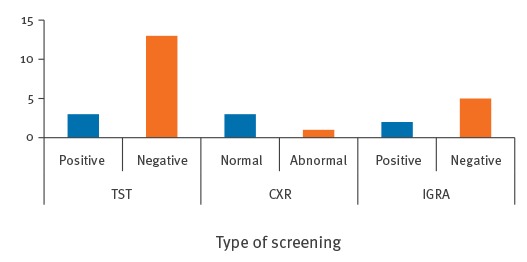
Results of tuberculosis screening by test type, contact investigations associated with air travel in Ireland, September 2011 to November 2014 (n = 23)

A total of 13 passengers had only TST performed, five had interferon-gamma release assay (IGRA) test performed and two passengers had both TST and chest X-ray. One passenger was screened with both IGRA and TST as the TST reading was 14mm; however, confirmatory results of the IGRA were negative. This passenger contact also had a bacillus Calmette-Guérin (BCG) scar. One contact was screened using IGRA and chest X-ray and the remaining passenger was screened by chest X-ray only. The screening results are outlined in Figure 2.

TST: Tuberculin skin test; CXR:  chest X-ray; IGRA: interferon-gamma release assay.

One passenger was clinically assessed for active tuberculosis only and was resident in a high-endemicity country and is not presented in this figure.

Some passenger contacts had more than one type of screening test performed.

### Screening results

Where results were available (n = 24) no active cases of TB were identified. Four passenger contacts were diagnosed with latent TB infection (LTBI). All four had other risk factors for LTBI. Two had travelled with the index case and were from a country of high endemicity. However, despite this, it was not possible to exclude transmission before air travel. The remaining two passenger contacts were also from a country of high endemicity, of whom one was also a healthcare worker.

## Discussion

This study investigated the possibility of *M*. *tuberculosis* transmission during air travel and found no evidence to support it. No active cases of TB were identified. Four passengers were diagnosed with LTBI; all were from countries of high-endemicity and one was a healthcare worker. In flight investigations it is often possible that passengers seated close to the index case may be family or friends. In this study, two passenger contacts diagnosed with LTBI had also travelled with the index case from a high-endemicity country and in this context it was not possible to determine whether transmission occurred during air travel or due to prior exposure. The inability of the TST to distinguish between recent vs earlier infection also contributes to this.

This study also identified challenges faced in obtaining complete timely airline manifests, which can lead to inadequate passenger information. Receipt of TB screening results from international colleagues was also challenging. Of all passenger contacts requiring TB screening, only 21 were identified as Irish citizens and 177 were international contacts. Of the 21 contacts screened in Ireland, results were received on 16, and of the 177 passenger contacts identified as international contacts, results were received on only eight contacts.

Interestingly, from published literature to date, no cases of active TB have been identified as a result of exposure to an infectious passenger during air travel and evidence suggests that few individuals infected with *M. tuberculosis* progress to active disease [18]. This study is consistent with previous contact investigations of TB during air travel, indicating that the risk of possible TB transmission is low. A large study conducted in 2010 in the US presented aggregated data from 131 index cases including 4,550 passenger contacts. This study identified that 182 (24%) had positive results and of the 142 passenger contacts with positive results for whom risk factor information was available, 130 (92%) had at least one risk factor and 12 (8%) had no risk factors. This study highlighted that positive TB test results were significantly associated with risk factors for prior TB [19]. This is reflected in our smaller study also.

A detailed UK systematic review [7] undertaken in 2010 reviewed 39 studies of which 13 were included in the review. This review found no evidence of transmission with only two studies reporting reliable evidence. The results also suggested reason to doubt the value of actively screening air passengers for infection with *M. tuberculosis* and recommended that the resources used might be better spent addressing other priorities in TB control. 

Based on currently available evidence, the risk of TB transmission during air travel is very low. A recent systematic review estimates the risk of TB transmission from a sputum smear-positive index case during air travel to be 0.1–1.3% [20]. 

In Ireland, current guidelines for the prevention and control of TB recommend contact tracing passengers as per the WHO guidelines, i.e. limited to the same row as the index case and two rows in front of and behind the index case. However, the updated risk assessment guidelines for infectious diseases transmitted on aircrafts (RAGIDA) by the European Centre for Disease Prevention and Control (ECDC) in 2014 [21] recommend considering additional criteria before commencing contact tracing of passengers during air travel. They advise contact tracing be commenced if the index case is confirmed with infectious pulmonary TB, and if there is evidence of transmission in other settings, such as transmission to household members or other close contacts. These guidelines suggest that where these criteria are met, exposed passengers in the relevant rows of the aircraft be contacted using the procedure outlined in the WHO guidelines. These RAGIDA guidelines also point out that in instances where (despite extensive efforts) no information on evidence of transmission to close contacts can be obtained, the national authority can nevertheless decide to initiate contact tracing in these exceptional circumstances. Investigating contact passengers using the 2014 RAGIDA criteria, however, could pose a challenge as in some instances the interval between case notification and identification of close contacts may be longer than anticipated due to various reasons, e.g. delays in locating contacts or delays in contacts presenting for screening. In such instances the 3-month interval as recommended in national and international air flight guidance between case notification to public health and date of flight may have elapsed.

Based on the available evidence on TB transmission during air travel, the National Institute for Health and Care Excellence (NICE) in the UK recommends that following a diagnosis of TB in an aircraft passenger, contact tracing of fellow passengers should not routinely be undertaken. They recommend that the consultant in communicable disease control (CCDC) provides the airline with ‘inform and advise' information to send to passengers seated in the same part of the aircraft as the index case [22].

Although there were limitations to this study, no cases of active TB were reported. This study was limited by the lack of comprehensive information from the airline manifests on each occasion. In total, 34 passengers had insufficient information available from the airline manifest and it was not possible to identify these passengers’ country of origin for screening. This limited the comprehensive follow-up on each exposed contact. Other limitations included the fact that only nine flights were eligible for follow-up, therefore further limiting the conclusions drawn from the study as over half of flights reported were not followed up. The incomplete receipt of TB screening results from international and national colleagues also limited the findings of this study.

Although information was available and TB screening requested on 198 passenger contacts, not all of these had sufficient information available, with some passengers only having nationality and passport numbers available from the airline manifest. As a result of this paucity of information, we cannot be certain how many of these passenger contacts were followed up as we received no further communication. Therefore, it was not possible to assess the effectiveness of TB contact tracing in these passengers.

The challenges faced in communicating with airlines and international colleagues regarding public health threats and subsequent interventions were highlighted in this study. The importance of improving communication between airlines and public health in relation to public health threats in general and improving the quality and timeliness of the data provided by airlines must remain a priority. This is particularly important due to the continuous emergence of new viruses and increased globalisation.

This study clearly highlights the difficulties and challenges experienced with TB contact tracing due to the poor quality of passenger contact information. This is not unique to Ireland with similar findings identified in a UK study which highlighted that the process of tracing and investigating contacts of air passengers infectious with TB is usually unsuccessful without the availability of appropriate contact information from the airlines [23].

This study also identified the challenges faced by public health in following up and screening contacts both nationally and internationally. As screening results were only obtained for 24 passengers, the possibility of more widespread transmission cannot be excluded. Contact tracing is time consuming and requires extensive resources. Questions in relation to the value of contact tracing passengers exposed to infectious TB during air travel were raised from this investigation and in relation to the possibility of more effectively re-allocating resources to other TB preventive and control activities. Consequently we recommend reviewing current Irish national policy in terms of routine contact tracing of passengers exposed to TB infection during air travel and exploring whether we should adapt the UK approach as outlined in the 2011 NICE guidance in terms of providing ‘inform and advise’ information only to passengers who have been exposed to TB on long-haul flights.

## Conclusion

Contact tracing has been used extensively in the prevention and control of TB. This retrospective review provided a unique opportunity to investigate the possibility of *M. tuberculosis* transmission during air travel. With an increase in flights to and from countries of high TB endemicity, the risk of passengers exposed to TB is inevitable, although our study found no evidence to support the transmission of *M. tuberculosis* from infectious passengers during air travel.

The issues surrounding incompleteness of data provided by airlines and also the lack of collaboration from airlines in providing airline manifests on request is of concern in this study and may have an impact on follow-up of other infectious diseases including those caused by emerging pathogens.
